# Magnetic mineralogy of the Baringo core (HSPDP-BTB13-1A, Kenya) shows astronomical forcing with implications for retrieving meaningful paleointensity

**DOI:** 10.12688/openreseurope.20266.1

**Published:** 2025-07-04

**Authors:** Mark J. SIER, Bianca R. Spiering, Mark J. Dekkers, Frits J. Hilgen

**Affiliations:** 1 Earth Sciences, Utrecht University Faculty of Geosciences, Utrecht, Utrecht, 3584CB, The Netherlands; 2 Centro Nacional de Investigacion sobre la Evolucion Humana, Burgos, Castile and León, Spain

**Keywords:** Chemeron Formation, East African Rift, Relative Paleo Intensity, Pliocene, Pleistocene

## Abstract

This study evaluates the potential of the BTB13 sedimentary core from the Baringo Basin, Kenya, to contribute to relative paleointensity (RPI) records and improve geochronological correlations across Eastern Africa. The core, spanning ~3.3-2.6 Ma, is part of the Hominin Sites Paleolakes Drilling Project (HSPDP) and has a robust age model based on magnetostratigraphy and 40Ar/39Ar dating. High-resolution rock magnetic analyses, including NRM/ARM ratios and pseudo-Thellier RPI methods, were applied to test the suitability of the core for RPI reconstruction. Results indicate that the magnetic signal is dominated by environmental rather than geomagnetic influences, with large amplitude NRM/ARM variations failing standard selection criteria for RPI. Spectral analysis reveals strong periodicities at ~22, ~40, ~50, and ~400 kyr, aligning with orbital parameters and suggesting that the magnetic signal is climatically driven, likely linked to lake level changes paced by precession and obliquity cycles. While unsuitable for RPI-based correlations, the BTB13 core preserves a valuable astrochronological signal that can support regional correlation and paleoenvironmental reconstruction in the context of hominin evolution.

## Introduction

Relative paleointensity (RPI) is a tool used to constrain the age of sedimentary sequences younger than 4 Ma ( Channell *et al*., 2018; Valet *et al*., 2020; Valet *et al*., 2005a), based on correlating short- and long-term fluctuations in the intensity of the Earth´s magnetic field (e.g., Sint2000; Valet *et al*., 2005b). Intensity changes in the Earth´s magnetic field occur at much higher frequencies than reversals of the Earth´s magnetic field. Like the Earth’s magnetic reversal record, the RPI record is irregular, unique in time, and the main trend is largely global, meaning that different regions reveal a similar pattern of high and low intensities for the same period of time (e.g. Channell *et al*., 2018; Tauxe, 1993). Therefore, the RPI from different localities can be compared and potentially correlated if the records are of similar age and of sufficient duration. The integration of RPI with other geochronological techniques, such as radioisotopic dating and magnetostratigraphy, allows for robust age models.

**Figure 1.**
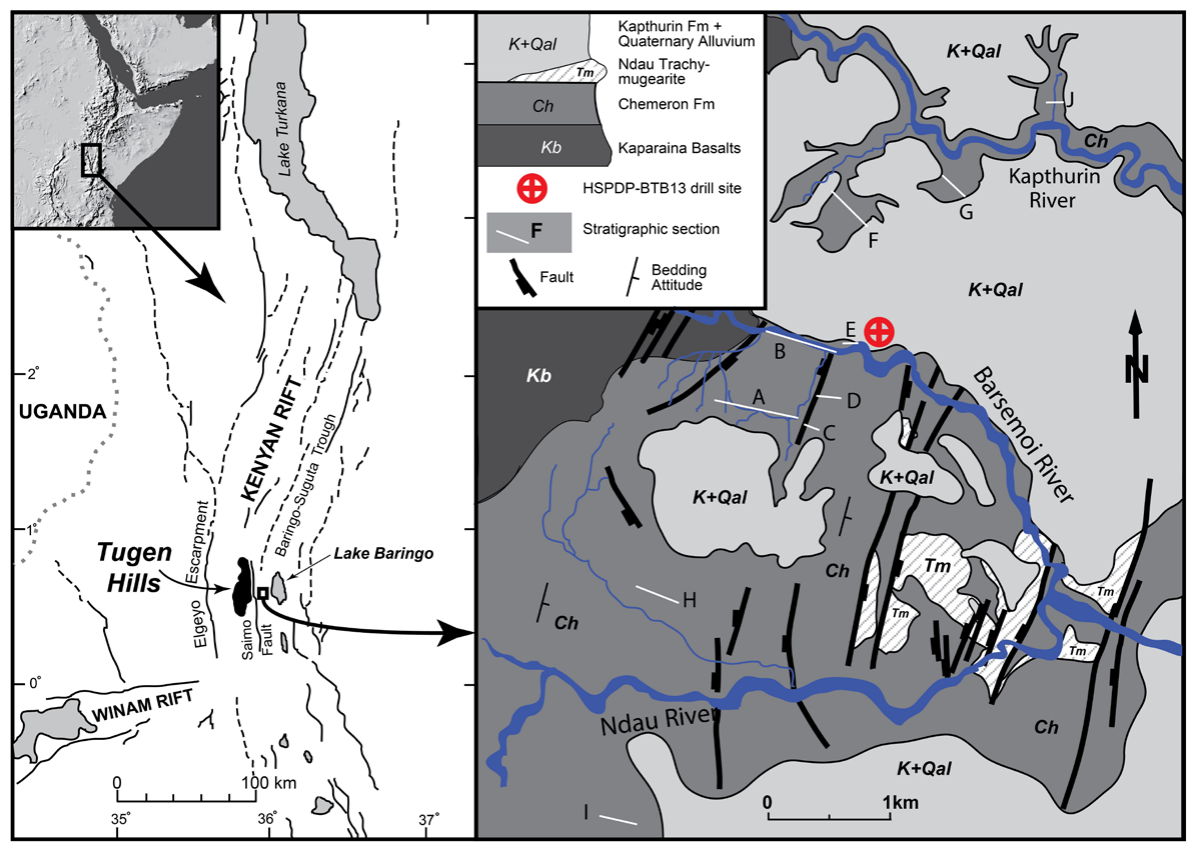
Map of the Baringo area with geological setting, top left insert shows topographic map of East Africa. Location of BTB13 site (latitude 0.5546N, longitude 35.9375E). Stratigraphy of this figure is described in detail in Deino *et al.*, 2006 and Kingston *et al.*, 2007. Figure adapted from Deino *et al.*, 2006.

The Hominin Sites Paleolakes Drilling Project (HSPDP) is an international multidisciplinary effort that aims to understand how landscape and climate change shaped the evolution of the hominin lineage ( http://hspdp.asu.edu/). Five key (paleo) lake areas were selected for HSPDP in Kenya end Ethiopia: Chew Bahir, Magadi, West Turkana, Northern Awash, and Baringo Tugen Hills Barsemoi (BTB13; Figure 1). Sediment cores were drilled at the selected sites that represent crucial areas for tracking human evolution in Eastern Africa. Evidently, age control is critical in such studies as it constitutes the basis for most of the major hypotheses in palaeoanthropology and archaeology. For the BTB13 sedimentary core from Kenya ( Figure 1) a high-resolution age model was published in 2021 based on dated tephras and magnetostratigraphy ( Deino *et al*., 2021; Sier *et al*., 2021). The geochronological well-constrained BTB13 core in principle can be used to generate an RPI record for future detailed time-stratigraphic correlations to other HSPDP cores, like the Northern Awash cores from the Afar region in Ethiopia, which are geochronologically poorly constrained, but overlap in age with the BTB13 core. Moreover, RPI data of the BTB13 core may contribute to the global RPI record, which remains inadequately documented for the age range represented by the BTB13 core. With this in mind, we measured rock magnetic properties of the BTB13 core. However, to achieve the objectives we first should test whether the samples are suited for RPI reconstruction by determining the origin of the paleomagnetic signal.

## Regional and geological setting

The BTB13 drill site (latitude 0.5546 ^◦^N, longitude 35.9375 ^◦^E) is located in the Baringo Basin/Tugen Hills, part of the Central Kenya Rift Valley. The drill site is located east of the Saimo Fault, west of Lake Baringo, and just north of the Barsemoi River ( Figure 1). The basin contains the most complete Late Neogene sequence of the East African Rift with a duration of 16 Myr ( Behrensmeyer *et al*., 2002; Chapman & Brook, 1978; Deino *et al*., 2002; Hill, 2002) and a thickness of ~3 km ( Gilbert *et al*., 2010). The strata have been divided into a number of formations and contain fluvio-lacustrine sediments that alternate with layers of trachyte, phonolite and basalts. The Pliocene/Pleistocene Chemeron Formation, the target of the BTB13 core, was first formally described by McCall *et al*. (1967) and ranges in age from 5.3 to 1.6 Ma ( Deino *et al*., 2006). The Chemeron Formation consists of lacustrine and subaerial sedimentary strata and some siliceous tuffs. It is exposed in the eastern foothills of a westward tilted horst block within the basin. A total of 228 meter of core was recovered ( Campisano *et al*., 2017) and the cored strata are correlated to dated outcrops ranging from ~2.6 to 3.3 Ma ( Cohen *et al*., 2016; Deino *et al*., 2021). The core is primarily composed of clastic deposits, which include a range of materials from muddy fine-grained sandstones to pebble conglomerates. These are interspersed with clayey and diatomaceous lacustrine deposits. The core can be divided into four main lithologic intervals ( Westover *et al*., 2021):

1. **Basal Interval:** This section, ranging from approximately 228 mbs (meters below surface) to 201 mbs, is primarily composed of conglomerates and mudstones.

2. **Finer-Grained Clastic Interval:** Extending from around 201 mbs to 107 mbs, this interval is dominated by fine-grained clastic deposits with occasional deep lacustrine layers.

3. **Conglomerate-Rich Interval:** From approximately 107 mbss to 78 mbs, this section is characterized by a high concentration of conglomerates.

4. **Upper Lacustrine Interval:** This interval, spanning from 78 mbs to the top of the core at 5 mbs (with the first 5 meters not recovered), consists of diatomaceous and clayey deep lacustrine sediments interbedded with fine-grained clastics.

The Upper Lacustrine Interval contains five cyclic deep-lake diatomites that are correlated to Diatomite 5 to Diatomite 1 from youngest to oldest in outcrop strata ( Deino *et al*., 2006; Kingston *et al*., 2007).

## Materials and methods

The BTB13 core was drilled in May-June 2013 (for drilling details see Cohen *et al*., 2016). After drilling, the BTB13 core was transported by airfreight to the National Lacustrine Core Facility (LacCore) at the University of Minnesota (Minneapolis, MN, USA) for comprehensive scanning, processing, description, and subsampling. Detailed analyses of physical properties of cores from all sites were conducted using a Multi-Sensor Core Logger - Standard (MSCL-S, for natural gamma radiation) and a MSCL-XYZ sensor (split core for high-resolution magnetic susceptibility) at intervals ranging from 0.5 to 4 cm. Gamma radiation values of 1 gm/cc (grams per cubic centimeter) and smaller were excluded from interpretation. Values of 1 gm/cc are consistent with water and values < 1 are deemed artifacts for example caused by scanning gaps. Visual core descriptions were also carried out (see Cohen *et al*., 2016 for more details). The paleomagnetic samples used in this study and our previous work ( Sier *et al*., 2021) were collected directly after the splitting of the core at the facility. 

A total of 543 levels were sampled for a total of 567 paleomagnetic samples at regular intervals throughout the core, with an average spatial resolution of around 0.41 m. Some levels were sampled twice with small unoriented samples collected for rock magnetic experiments. The stratigraphic position of the samples was measured with < 0.5 cm precision. Several methods were used to collect samples. The majority were extracted using a drill press equipped with a water-cooled diamond-coated 2.5 cm diameter bit. Soft levels were sampled by gently pushing custom-made quartz or perspex containers, with standard paleomagnetic sampling dimensions (25 mm diameter, 22 mm length), into the sediment. Levels that were too fragile for drilling but not soft enough for insertion of the quartz cups or perspex containers were carefully carved out in blocks of 2×2 cm. All lithologies encountered in the core were sampled; intervals where the core was distorted as a result of the drilling were avoided, these were mainly situated at the start of core segments. After sampling, the samples were wrapped in laboratory-grade airtight cellophane and stored at temperatures below 5° C.

For our pilot RPI study we performed stepwise progressive static 3-axis Alternating Field (AF) demagnetization of the NRM up to a maximum of 100 mT, in 16 steps using the integrated AF demagnetizer of the DC-SQUID magnetometer at the Centro Nacional de Investigacion sobre la Evolucion Humana (CENIEH) in Burgos (Spain). After each step the remaining NRM was measured with a 2G DC-SQUID magnetometer. NRM intensities were typically several orders of magnitude higher than the instrument sensitivity (~3×10 ^−12^ Am ^2^). During the demagnetization process, the specimens were kept in a shielded environment. Stepwise. Anhysteretic Remanent Magnetisation (30 µT steady field) (ARM) acquisition was performed with the same equipment as mentioned above using the same 16 steps. For the pilot data set on 40 randomly selected samples so-called Pseudothellier analysis ( Tauxe *et al*., 1995) was performed using the Paleointensity.org open source, online tool for analyzing paleointensity data ( Béguin *et al*., 2020). Interpretation was done using the automatic interpretation option on specimen level where only samples that pass the set of selection criteria are considered. These selection criteria are:

n≥5, f≥0.35, β≤0.1, k ^→^' abs≤ 0.164, R ^2^corr ≥0.995, MAD _Free _≤ 5 |k ^→^′|≤0.2 f _Resid _<0.15, α ≤ 5, DANG ≤ 4, k ^→^' _AA_ abs≤ 0.2, k ^→^' _DD_ abs≤ 0.2, R ^2^_corr_ _AA_ ≥ 0.995, R ^2^_corr DD_ ≥ 0.995, f _Resid_ ≤ 0.15 ( Béguin *et al*., 2020; Paterson *et al*., 2016). A second automatic interpretation was done with the AF steps up to 15 mT omitted, as our previous work indicated an overprint in these samples up to 15 mT ( Sier *et al*., 2021). The remaining samples were measured using the methods above with the exception of the ARM only being measured at 25, 30, and 35 mT. As a result, the data cannot be used for pseudo Thellier RPI but it can be used as a proxy for Magnetic Mineral Content (NRM/ARM) and shows distinct regularity leading us to investigate whether this variability is related to astronomical climate forcing.

### Age model

The age model used is in our study is a Bayesian stratigraphic age model based on four identified paleomagnetic reversals in the core ( Sier *et al*., 2021), ^40^Ar/ ^39^Ar dating ( Deino *et al*., 2021), and ^40^Ar/ ^39^Ar age calibration of related outcrop diatomaceous units ( Deino *et al*., 2006; Kingston *et al*., 2007). ^40^Ar/ ^39^Ar dating was done on 12 tephras from the core and 4 tephras in outcrop that were geochemically correlated to the core. The age model of the BTB13 core has not been astronomically tuned, however, the diatomaceous units near the top of the core have been previously linked to precession cycles ( Deino *et al*., 2006; Kingston *et al*., 2007). Uncertainties of the age model vary throughout the core, with the lowest uncertainties in the upper part that contains most geochronological tie points (see figure 6 of Deino *et al*., 2021).

### Spectral analysis

Spectral analysis of the NRM/ARM data was performed with the open source software package Acycle ( Li *et al*., 2019). The data were analyzed in the time domain using the age model of Deino *et al*. (2021). The data were stratigraphically sorted, interpolated to a uniform sample spacing equal to the median sample distance (~0.34 m and ~1 kyr), and demeaned so that it varies around a mean of zero. Subsequently, periodicity was evaluated by applying the multitaper method with default settings (i.e., three 2π prolate tapers with no zero-padding) ( Thomson, 1982). Confidence levels were acquired based on the classic first‐order autoregressive (AR1) Red Noise model ( Gilman *et al*., 1963; Husson, 2014). Bandpass filtering was applied using the Gauss algorithm ( Kodama & Hinnov, 2015) to isolate specific frequency bands.

## Results

### Relative paleointensity and magnetic mineral concentration

**Figure 2.**
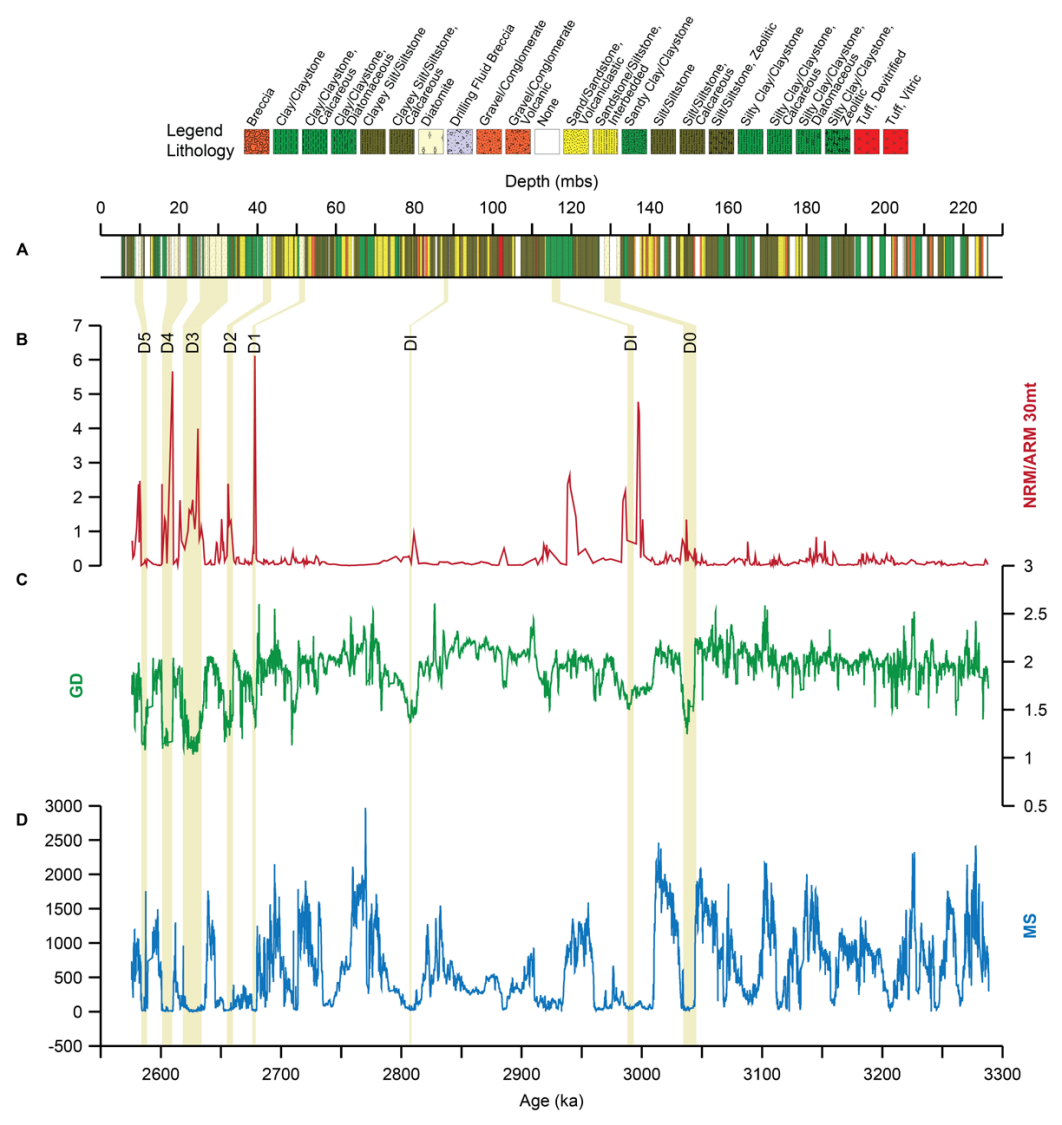
Overview of the BTB13 core data. Lithological log of the BTB13 core ( **A**), NRM/ARM 30 mT NRM/ARM (red; **B**), Gamma Density (GD; green; g/cm ^3^; **C**), and magnetic susceptibility (MS; blue; 10 ^-5^; **D**). The position of the diatomites (D0-D5) and diatomite intervals (DIs) is indicated by the beige vertical bars.

The pilot study of RPI using pseudo-Thellier (see SI, Data availability) shows that only around 50 % of the samples pass the default auto selection criteria of Paleointensity.org (see Béguin *et al*., 2020; Paterson *et al*., 2014, for details) and only 1 sample passes when AF demagnetization steps up to 15 mT are deleted. In Figure 2 the BTB13 record of NRM/ARM at 30 mT (487 samples) has been plotted against age together with lithology, gamma density records (GD) and magnetic susceptibility (MS). The BTB13 NRM/ARM data varies several orders of magnitude: maximum values are around 250 times larger than the minimum values with well-defined peaks ( Figure 2). Some of these peaks, but not all, correspond with the diatom levels described in the core ( Figure 2), which in turn correspond to minima in the GD and MS. The NRM/ARM peaks are highest between ~2.57 and ~2.7 Ma, where the five diatomites occur, and ~2.93 and 3.05 Ma.

### Cyclostratigraphy and time series analysis

**Figure 3.**
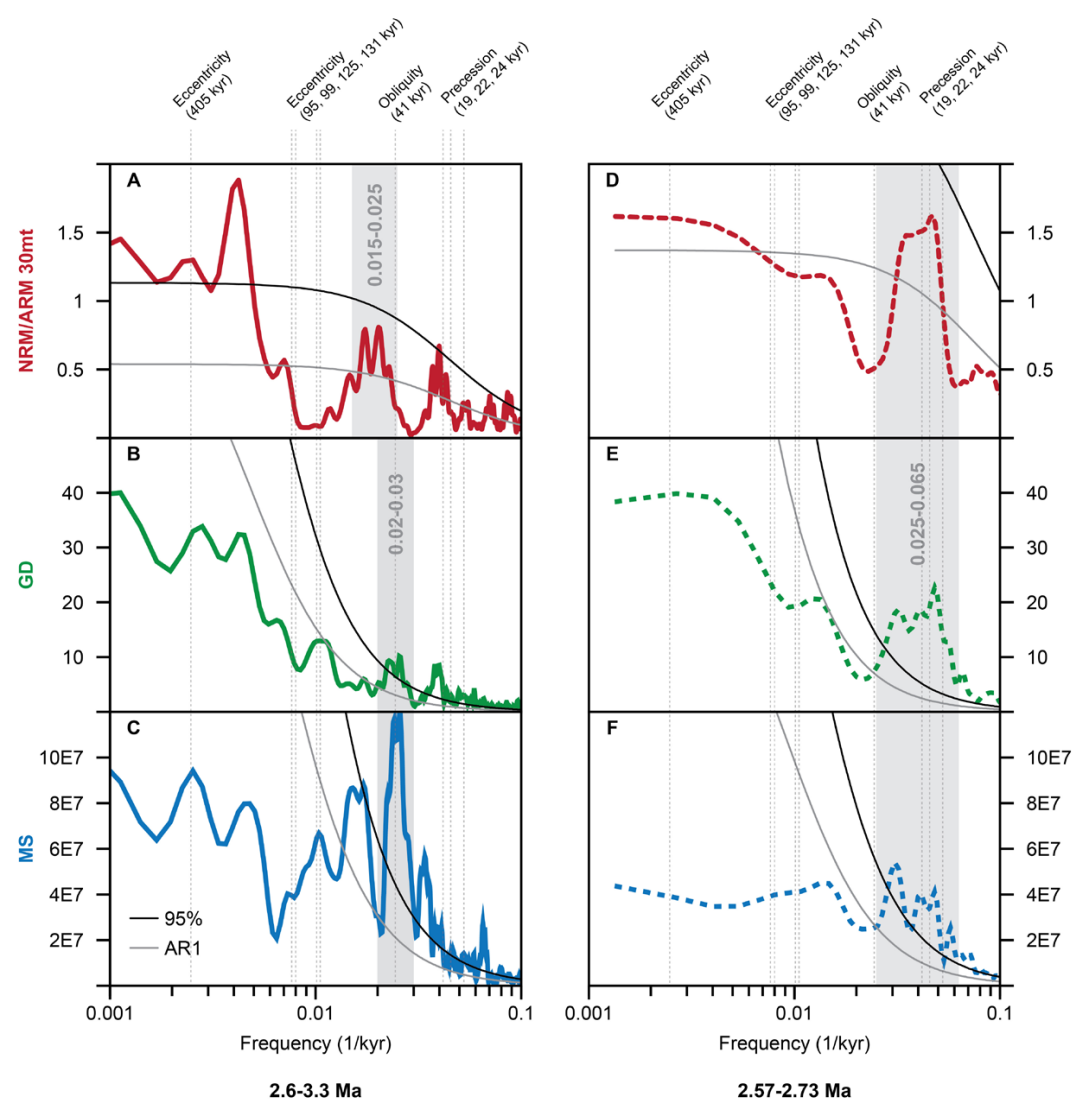
Spectral analysis of the BTB13 core data in the time domain for the 2.6–3.3 Ma (0–230 mbs) interval (solid lines; **A**- **C**) and the 2.57–2.73 Ma (0–75 mbs) interval (dashed lines; **D**- **F**). NRM/ARM in red, GD in green, and MS in blue. A log scale is used for the x (frequency) axis, and a linear scale is used for the y (power) axis. Grey and black solid lines indicate the AR1 model and 95% confidence level, respectively. The gray bars indicate important frequency bands centered around 0.02 1/kyr (50 kyr),0.025 1/kyr (40 kyr), and 0.045 1/kyr (22 kyr). The black dotted vertical lines indicate the main astronomical frequencies of eccentricity, obliquity, and precession.

**Figure 4.**
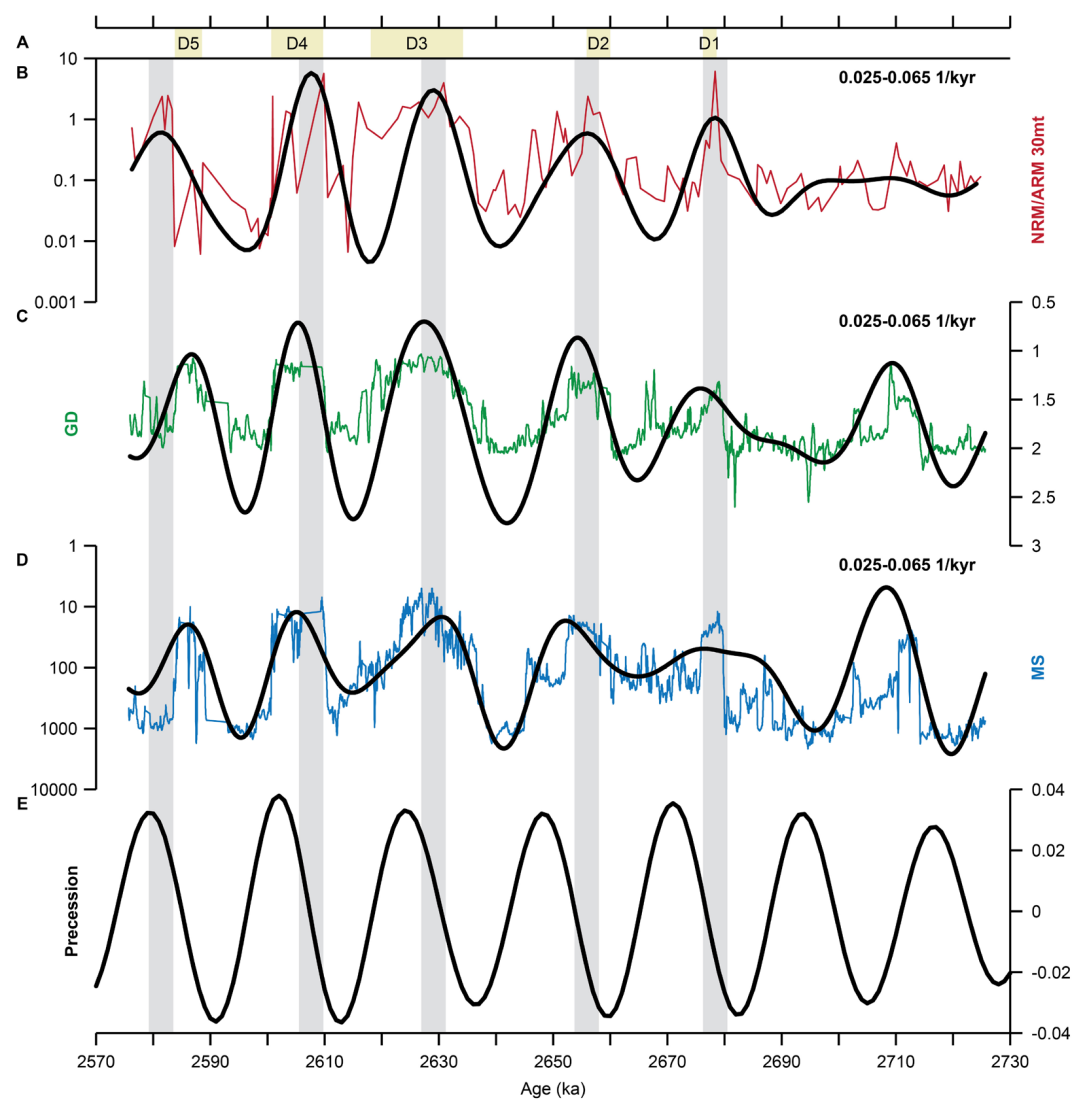
Bandpass filtering of the BTB13 core data for the 2.57–2.73 Ma (0–75 mbs) interval. NRM/ARM in red ( **B**), GD in green ( **C**), and MS in blue ( **D**). The filtered ~22 kyr cyclicity is shown in black (bandpass filter width of 0.025–0.065 1/kyr). Note that NRM/ARM and MS are plotted on a log scale, and MS and GD values are plotted reversed. The positions of the diatomites (D1-D5) are indicated by the beige bars at the top ( **A**). Grey vertical bars indicate major NRM/ARM filter maxima. The bandpass filters are compared to the La2004 precession solution ( Laskar *et al*., 2004; E).

In the time domain (~3.29–2.56 Ma), high spectral power occurs in three frequency bands centered on periods of ~22 kyr for the NRM/ARM, GD, and MS records, ~40 kyr for the GD and MS records, and ~50 kyr and ~400 kyr for NRM/ARM ( Figure 3 A-C). The 40 kyr cyclicity is particularly strong (>>95% confidence level) in the MS data. The 22 kyr cyclicity is mainly developed 2.57-2.73 Ma interval of the BTB13 core, with a stronger expression in the GD and MS data ( Figure 3 D-F). The ~22 kyr variability is consistent between the three datasets in the 2.57-2.73 Ma interval ( Figure 4): the NRM/ARM maxima concur with the GD and MS minima and with the diatomites.

**Figure 5.**
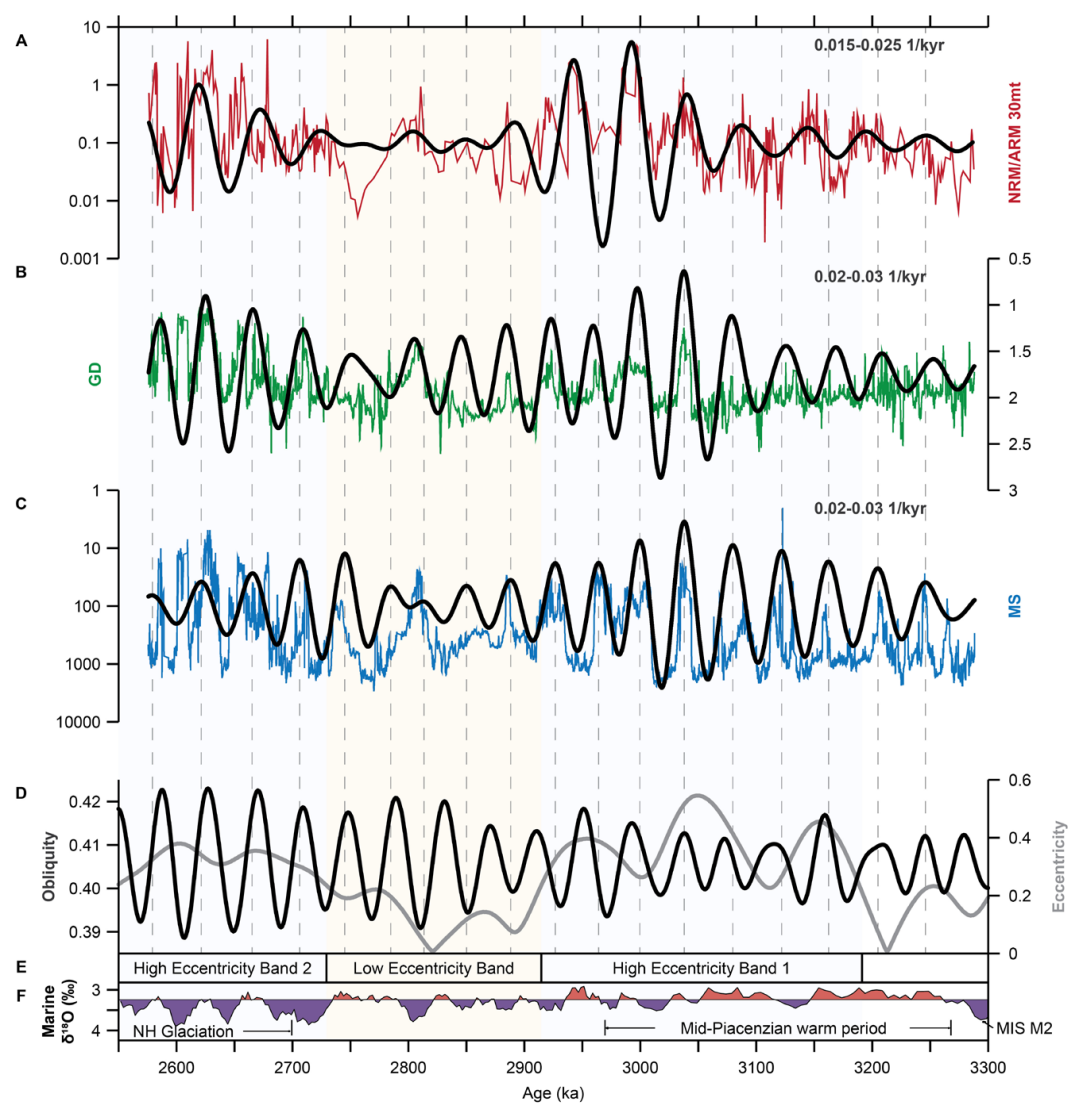
Bandpass filtering of the BTB13 core data for the 2.6–3.3 Ma (0–230 mbs) interval. NRM/ARM in red ( **A**), GD in green ( **B**), and MS in blue ( **C**). The filtered ~50 and ~40 kyr cyclicities are shown in black (bandpass filter widths of 0.015–0.025 and 0.02–0.03 1/kyr, respectively). Note that NRM/ARM and MS are plotted on a log scale, and MS and GD values are plotted reversed. Grey vertical dashed lines indicate MS filter minima of the ~40 kyr cyclicity. The bandpass filters are compared to the La2004 obliquity and eccentricity solutions ( Laskar *et al*., 2004; **D**). Intervals of high and low eccentricity are indicated ( **E**). LR04 marine benthic δ ^18^O stack ( Lisiecki & Raymo, 2005) is shown as a proxy for global ice volume ( **F**).

The ~50 kyr variability in the NRM/ARM data is roughly consistent with the ~40 kyr variability of the GD and MS datasets ( Figure 5a–c), while the ~40 kyr variability in the GD and MS datasets is consistent with one another ( Figure 5b, c). The filtered ~40 kyr cyclicity in the MS and GD data follows the obliquity solution (La2004) well ( Figure 5D). The ~400 kyr cyclicity in the NRM/ARM and GD data follows the long eccentricity cycle, with the exception that the filter maximum in the middle part of the core occurs slightly later than the long eccentricity maximum ( Figure 5D).

## Discussion and conclusions

### Do the RPI/NRM/ARM data reflect relative paleo-intensity?

Our pilot study using the pseudo-Thellier method demonstrates that the RPI data of the Baringo core are not suitable for reconstructing relative paleo-intensity (see SI for data). Only half of the 40 pilot samples meet the selection criteria of paleointensity.org, and when steps up to 15 mT are excluded—previously identified as the maximum AF step for low-coercivity overprint ( Sier *et al*., 2021) —only one sample qualifies. Another factor is that the depth profile of the 30 mT NRM/ARM data reveals intensity peaks significantly exceeding the range of values for a reliable RPI record. Intensity variations should be limited to a factor of 2–3 ( Tauxe, 1993), with some suggesting that a 25% variation is more appropriate ( Selkin & Tauxe, 2000).

Previous studies identified Ti-rich titanomagnetite as the dominant remanence carrier ( Deino *et al*., 2006), a finding later confirmed through rock magnetic analyses ( Sier *et al*., 2021). The remanence carrier predominantly falls within the single-domain (SD) range or, in some cases, near the superparamagnetic (SP) domain ( Sier *et al*., 2021). Although these boundary conditions are generally favorable for RPI studies ( Tauxe, 1993), the excessive variations in intensity and lack of samples passing the minimum selection criteria prevent the use of either the pseudo-Thellier or classic NRM/ARM RPI methods. Thus interpretation of the data in terms of RPI is ungrounded. The observed regularity and link to diatomites 1–5 already provide valuable clues about this origin, as they suggest that the variability is cyclic and related to astronomical climate forcing with which we will continue the discussion.

### Cyclicity

The “RPI”, GD, and MS records display significant cyclicity with periods that are close to those of the main astronomical cycles ( Figure 3). Variations occur with periods of ~22 kyr (in particular in the top of the core between 2.73 and 2.57 Ma) and ~40 kyr, suggesting that these cyclic variations are related to climatic precession and obliquity ( Figure 4 and Figure 5). There is a strong obliquity signal, but weaker expression of precession, in the pre-2.7 Ma part of the record, which has also been noted by Mitsunaga *et al*. (2023). However, in the top part of the core there is a dominance of precession. Here, diatomites are intercalated at a precession pacing and associated with NRM/ARM maxima and GD and MS minima ( Figure 2). It has been shown that these diatomites, and therefore GD and MS minima and RPI maxima, represent deep lake conditions ( Deino *et al*., 2006). Deino *et al*. (2006) also related the diatomites to precession, and more specifically to monsoonal intensification during boreal summer insolation maxima (i.e., precession minima), suggesting that the main driver of the observed lake level changes was the North African monsoon. This configuration results in maximum amounts of rainfall over the Sahara and Sahel and would result in increased river runoff towards the BTB13 site and subsequent deepening of the lake. Indeed, modelling studies performed by Bosmans *et al*. (2015) indicated a large increase in rainfall on the northern hemisphere African continent, including **e**astern Africa, during precession minima. The ~22 kyr variability in the data also neatly follows the precession cycle in the La2004 solution, with NRM/ARM filter maxima occurring at similar positions in a precession cycle ( Figure 4d). It is remarkable that this inferred phase relation is largely consistent with the phasing of the filtered 22-kyr cyclicity in the proxy records in the top part of the core relative to climatic precession. Although this could be a coincidence considering the uncertainties in the age model, it is worth mentioning that the Ar/Ar astronomically-tuned ages, which generally have small uncertainties, have been used to construct the age model. The Ar/Ar sanidine ages have been calculated relative to the astronomically calibrated age of 28.201 ± 0.046 Ma of Kuiper *et al*. (2008) for the Fish Canyon sanidine or to sanidine phenocrysts from the Alder Creek Rhyolite of California with an astronomical calibrated age of 1.1848 ± 0.0006 Ma ( Niespolo *et al*., 2017). In addition, astronomical ages of magnetic reversal boundaries have been used to construct the age model.

Evidently, the NRM/ARM record is strongly influenced by an astronomically-paced climatic signal, implying that it does not reflect variations in Earth’s magnetic field and thus cannot be used for correlations to other cores using relative paleo-intensity as a tool. However, the astronomical cyclicity in the RPI/NRM/ARM, GD and MS records provides alternative opportunities for correlating the Baringo core to other African lake records with poor age control, like the Northern Awash cores. In addition, these records can potentially be used to construct an astrochronological framework based on tuning the recorded cyclicity in the records to astronomical target curves (e.g., Hilgen *et al*., 2022; Joordens *et al*., 2011). For future work on RPI/NRM/ARM records from sedimentary records we believe that, regardless of the quality of RPI/NRM/ARM records, it is important to include spectral analyses as an quality indicator as it provides a straightforward method for identifying cyclicity, which should be absent in high-quality RPI records.

### Correlation to other regional continental cores

Like the diatomites, large NRM/ARM peaks only occur during 405 kyr eccentricity maxima, referred to as high eccentricity intervals ( Figure 5E). The NRM/ARM peaks between 2.7 and 2.58 Ma are centered at a 405 kyr eccentricity maximum (see Figure 5), while the NRM/ARM peaks between 3.2 and 2.92 Ma occur slightly after an eccentricity maximum. This delay resulted in a periodicity of ~360 kyr, which is somewhat shorter than the 405 kyr period of the long eccentricity cycle. All diatomites correspond to NRM/ARM peaks in our record, but not all of the measured NRM/ARM peaks are associated with a diatomite ( Figure 2). This suggests that the NRM/ARM peaks are not an expression of the diatomite formation, but that both the diatomite formation and the NRM/ARM peaks are an expression of the same driving force. This is important for the correlation between the Northern Awash and Baringo cores. Although no diatomites have been identified in the Awash cores, it is possible that future study of the rock magnetic data will show a similar pattern as in the Baringo core, allowing for the identification of intervals of high and low eccentricity on the long 405-kyr eccentricity scale ( Figure 5E). If so, this NRM/ARM, GD, and MS data could still help with building a geochronological model for the Northern Awash cores.

For future research on RPI records derived from sedimentary sequences, we recommend incorporating spectral analyses regardless of the perceived quality of the records. Spectral analysis offers a straightforward method for detecting cyclicity, which should be absent in high-quality RPI records according to most workers. Additionally, NRM/ARM records hold potential for chronological applications, particularly if they can be reliably correlated with astronomical cycles.

## Ethics and consent

Ethical approval and consent were not required.

## Data Availability

The project contains the following underlying data published on figshare.com: BTB pilot set NRM/ARM data: https://doi.org/10.6084/m9.figshare.28904759.v1 Paleonintensity.org export data: https://doi.org/10.6084/m9.figshare.28906790.v1 BTB NRM/ARM data: https://doi.org/10.6084/m9.figshare.28905680.v1 Data are available under the terms of the Creative Commons Attribution 4.0 International license (CC-BY 4.0).
