# Supplementary material for: Magnetic mineralogy of the Baringo core (HSPDP-BTB13-1A, Kenya) shows astronomical forcing with implications for retrieving meaningful paleointensity
Source: Open Res Eur. 2025 Jul 4;5:177. [Version 1] doi: 10.12688/openreseurope.20266.1 (PMC12756590; doi:10.12688/openreseurope.20266.1)
Supplement: Peer review history

# Reviewer report: Reviewer response for version 1

**DOI:** 10.21956/openreseurope.21927.r64543

**Published:** 2026-01-09

**Authors:** Tilo von Dobeneck

**Affiliations:** 1 University of Bremen, Bremen, Germany

Tilo von Dobeneck

 University of Bremen, Faculty of Geosciences and MARUM Research Center, Germany

 Open review of the Open Research Europe research article “Magnetic mineralogy of the Baringo core (HSPDP-BTB13-1A, Kenya) shows astronomical forcing with implications for retrieving meaningful paleointensity” by M.J. Sier, B.R. Spiering, M.J. Dekkers & F.J. Hilgen

 Following up on the available open-access review by Tesfaye Kidane (2025), I also see the interest of this third in a series of chronostratigraphic papers on Baringo-Tugen Hills-Barsemoi core BTB13-1A, starting with 40Ar/39Ar and magnetostratigraphic dating by Deino et al. (2021), supported by a more detailed presentation and rock-magnetic backup of the paleomagnetic results by Sier et al. (2021), and now expanded to cyclostratigraphy and RPI methodology by Sier et al. (2025). Despite the large petrological and sedimentological inhomogeneity of the sediment core, the time series analysis of physical property logs of magnetic susceptibility (MS) and gamma density (GD) – and to some surprise – even of the supposedly chaotic NRM/ARM “RPI” signal, carry significant spectral energy in the obliquity and eccentricity band. This unexpected fact (but less the initial aim for the laborious RPI analysis to correlate NRM/ARM to RPI stacks) merits to present, filter and interpret all three paleorecords in this third paper and to investigate the astronomical forcing of NRM/ARM, MS and GD alongside.

 The paper makes various general and partly ambivalent statements on the nature and applicability of the “Relative Paleointensity” (RPI) method, which could be more differentiated for better precision. In the starting Introduction sentence, RPI is being introduced as a “tool used to constrain the age of sedimentary sequences younger than 4 Ma, based on correlating short- and long-term fluctuations in the intensity of the Earth´s magnetic field”. The RPI method has no principal (physical) age limitations such as for example the radiocarbon method. However, for sedimentological and technical reasons, it is indeed difficult to identify and continuously core and u channel sample (Valet et al. 2020) older marine sediments with reliable preservation of paleofield intensity. To my knowledge, the longest continuous RPI record at present is that of Pacific IODP Site U1335 (Yamazaki & Yamamoto, 2018), which goes back to 8 Ma. Satisfactory RPI recording qualities of some abyssal cores (e.g. Wang et al. 2021) signalize, that it may be possible in future to extend the reach of RPI stacks further back in time with dedicated coring, but certainly never anywhere like the Geomagnetic Polarity timescale.

 Which matters more to me, is the paper's problematic equalization of RPI (1) as “tool” (line 1, paragraph 1) for chronostratigraphic correlation (assuming its validity!), (2) as a paleomagnetic ratio parameter (NRM/ARM), and (3) as reconstruction of “fluctuations in the intensity of the Earth’s magnetic field”. We may agree that in the ideal case of homogenous sediments with stable accumulation conditions, constant texture and uniform, unaltered fine-grained magnetic carriers, RPI does provide a credible proxy record of millennial-scale geomagnetic field intensity changes.

 In the more common case of heterogeneous sediments deposited under changing environmental and sedimentological conditions (such as HSPDP-BTB13-1A), geomagnetic field intensity changes are just one (and as it seems here, a minor one) of several factors controlling the ARM-normalized NRM intensity record. Calling the BTB13 NRM/ARM record a “RPI record” sounds therefore euphemistic. The authors’ initial goal (Abstract lines 9-10) of “testing the suitability of the core for RPI reconstruction” is a test, that was bound to fail given the rather noisy NRM inclination and declination data and diverting rock magnetic characteristics presented by Sier et al. (2021). It appears more reasonable to depart from the goal of analyzing the characteristics and possible expression of the NRM/ARM ratio in a complex continental (lacustrine/eolian) sedimentary sequence (which most of the paper actually does) than to aim for a valid, continent-wide applicable RPI reconstruction.

 The authors try to resolve this controversy on page 8 by introducing the term “RPI/NRM/ARM”, which I consider as unfortunate, since the first dash symbolizes “and/or” while the second simply means “:”; this terminology is both mathematically and formally confusing. The article title also reads somewhat too euphemistic, since “… with implications for retrieving meaningful paleointensity” (foreseeably) translates more realistically into “…. and causes failure of the RPI method”. I admit that a “trial-and-error” approach occasionally beats applicability rules of chronostratigraphic methods, but alone the lithostratigraphic description of the core casts enormous doubt on the promise of obtaining a RPI record in sensu stricto: interbedded “muddy fine-grained sandstones to pebble conglomerates”, tephras and diatomites, with soft layers, that could be sampled with perspex push-cubes, and hard layers needing diamond-bit drilling; a greater sediment hetereogeneity is hardly imaginable!

 Proposing RPI as a potential chronostratigraphic “tool” to refine a (in my impression) very noisy and quite poorly defined directional paleomagnetic record through correlation to RPI stacks appears to me as overoptimistic and misleading approach. This sends a wrong signal to the paleochronology community, who then approaches us paleomagnetists to “kindly RPI-date” all their otherwise “undatable” non-fossiliferous clastic sediment cores. In agreement with the first reviewer, I suggest to reformulate this first article version giving priority to the investigation of orbital cyclicity of rock magnetic records, including the question, which lithological attributes and driving environmental factors control NRM/ARM.

 In the first place (this should be more clearly stated!), NRM/ARM measures the grain-level magnetization times the degree of joint spatial magnetic orientation (or randomization) of fine magnetic particles within the sedimentary matrix – which may or may not reflect paleofield intensity. Studies by Franke et al. (2004) and Hofmann & Fabian (2007, 2009) have statistically quantified the impact of magnetic grain-size, clay mineralogy, carbonate and diatom content, alteration and other sedimentary factors of NRM/ARM and demonstrated, that “RPI” records usually carry a relevant environmental overprint.

 Given that the bandpass-filtered signals of the high-resolution MS and GD records largely agree and positively correlate with equally filtered, but interpolated, low-resolution NRM/ARM-derived “RPI” signal indeed implies, that the two-orders-of-magnitude variations of MS entail a proportional, also two-orders-of-magnitude (and therefore definitely not geodynamo-like) variability of NRM/ARM. It would be interesting to see here as well the bandpass-filtered signals and frequency spectra of the separate NRM and ARM records to elucidate, which of the two parameters carries more of an orbital signature (probably ARM), which it inherits to the NRM/ARM ratio parameter. If both NRM and ARM were equally affected by Milancovic cycles, the climatic influence should cancel out in their ratio. It may be useful to also have a correlation matrix of log(MS), GD, log(ARM), log (NRM), log(NRM/ARM), ARM/IRM (or log(ARM/IRM), if normally distributed) together with respective eccentricity, obliquity and precession or insolation target curves. Cross-and phase spectral analyses could eventually deliver orbital characteristics of interesting parameter correlations.

 A minor flaw is the use of two different density units, where “gm/cc” is used twice in the text and “g/cm³” in the Figure 2 legend - only the latter is a valid SI unit. In terms of style and language, figure quality and statistical methodology, the manuscript fulfills modern indexing standards. The article layout would however greatly profit from allowing Figures 1, 2, 3 and 5 to occupy as much lateral space as Figure 4, which only commands over sufficiently large (amongst others, font) size; for the readability of detailed illustrations, particularly in paper printouts, size definitely matters!

Is the study design appropriate and does the work have academic merit?

Partly

Is the work clearly and accurately presented and does it cite the current literature?

Partly

If applicable, is the statistical analysis and its interpretation appropriate?

Partly

Are all the source data underlying the results available to ensure full reproducibility?

Yes

Are the conclusions drawn adequately supported by the results?

Partly

Are sufficient details of methods and analysis provided to allow replication by others?

Yes

Reviewer Expertise:

Environmental and Rock Magnetism, Magnetostratigraphy, Sediment Physical Properties, Paleoceanography, Geostatistics

I confirm that I have read this submission and believe that I have an appropriate level of expertise to confirm that it is of an acceptable scientific standard, however I have significant reservations, as outlined above.

## References

[1] : Does lithology influence relative paleointensity records? a statistical analysis on South Atlantic pelagic sediments. *Physics of the Earth and Planetary Interiors* .2004;147(2-3) : 10.1016/j.pepi.2004.07.004 285-296 10.1016/j.pepi.2004.07.004

[2] : Rock magnetic properties and relative paleointensity stack for the last 300 ka based on a stratigraphic network from the subtropical and subantarctic South Atlantic. *Earth and Planetary Science Letters* .2007;260(1-2) : 10.1016/j.epsl.2007.05.042 297-312 10.1016/j.epsl.2007.05.042

[3] : Correcting relative paleointensity records for variations in sediment composition: Results from a South Atlantic stratigraphic network. *Earth and Planetary Science Letters* .2009;284(1-2) : 10.1016/j.epsl.2009.03.043 34-43 10.1016/j.epsl.2009.03.043

[4] : Dating North Pacific Abyssal Sediments by Geomagnetic Paleointensity: Implications of Magnetization Carriers, Plio-Pleistocene Climate Change, and Benthic Redox Conditions. *Frontiers in Earth Science* .2021;9: 10.3389/feart.2021.683177 10.3389/feart.2021.683177

[5] : Relative Paleointensity and Inclination Anomaly Over the Last 8 Myr Obtained From the Integrated Ocean Drilling Program Site U1335 Sediments in the Eastern Equatorial Pacific. *Journal of Geophysical Research: Solid Earth* .2018;123(9) : 10.1029/2018JB016209 7305-7320 10.1029/2018JB016209

# Reviewer report: Reviewer response for version 1

**DOI:** 10.21956/openreseurope.21927.r64548

**Published:** 2025-12-31

**Authors:** Julie Carlut

**Affiliations:** 1 Université Paris Cité, Institut de Physique du Globe de Paris, Paris, France

The topic of this study is very interesting. The astronomical cyclicity identified in the NRM/ARM, Gamma Density (GD), and especially in magnetic susceptibility suggests that this core could potentially be used to constrain other African lake records that suffer from poor age control. This is particularly valuable given that the BTB13 core spans a time interval overlapping with key hominin-bearing horizons in the region.

 Several aspects would benefit from deeper discussion. One point is that the authors do not fully embrace the production of a magnetic mineralogy study. This is likely because the initial objective: reconstructing relative paleointensity variations and establishing a robust age model, was difficult to achieve. Nevertheless, the dataset presented contains interesting and potentially valuable magnetic results. A related difficulty is that the study ends up being highly dependent on an age model that is itself incomplete, since constraining age models was a goal of the work. This circularity can give the overall impression of a somewhat fragile framework, which could be alleviated by a more explicit (an additional figure?) presentation of the age model based on the 4 reversals and twelve 40Ar/39Ar ages and its uncertainties/limitations.

 Minor points

 In the Introduction, the first paragraph would be better placed at the end of the section and shortened. Indeed, this paper is not truly a relative paleointensity (RPI) study in the classical sense, but rather a study emphasizing variations in magnetic properties more broadly.

 In the Geological Setting section, it is clear that the sedimentary sequence is highly heterogeneous, including lithologies that are particularly unfavorable (e.g., conglomerates). It is well known that paleointensity studies are generally discouraged in heterogeneous sections (see, for example, Tauxe et al., 1993), and therefore disappointing results were to some extent predictable. In this respect, I do not fully agree with the authors’ statement referring to “favorable magnetic carriers,” as the strong lithological heterogeneity is not sufficiently acknowledged. Ultimately the cyclostratigraphic signal identified here could be already expressed in the lithology itself, something that the Gamma Density proxy partly captures.

 The Material and method section contains details that are not really relevant (as “the core was transported by airfreight…”)

 In the Age Model section, it would be beneficial to introduce upfront the elements that are currently discussed later (page 8). Given how critical the age model is to the entire study, it clearly requires clarification from the beginning. Regarding the spectral analyses, the interpolation of the data to obtain a uniform 1 kyr sampling is a potentially critical step. It is important to ensure that this procedure does not, in any way, condition or bias the results of the spectral analysis. The ~400 kyr cycle appears rather unconvincing to me: the signal is weak and the number of observed cycles is limited.

 Ultimately, magnetic susceptibility stands out as the parameter carrying the most robust and interesting signal. But I regret that only NRM/ARM data are presented. Including more classical rock magnetic proxies, such as grain-size indicators (e.g., ARM/K), would strengthen the interpretations.

 The zoomed-in analysis over the 2.73–2.56 Ma interval is quite interesting. The correlation between diatomite and precession minima is moderate (three cases out of five), but once again uncertainties in the age model may explain this imperfect correspondence. Phase offsets are observed between maxima in NRM/ARM (and magnetic susceptibility) and the precession signal. The signal appears rich and complex; understanding the origin of these phase relationships would likely be extremely informative. Finally, Figure 5 is particularly interesting and could benefit from a more thorough discussion.

**Overall**, the authors convincingly demonstrate the presence of a useful astrochronological signal revealed by magnetic parameters in the studied section. The age model it is based on should be more explicitly shown. A figure showing ARM alone and/or ARM/K might be a valuable addition too.

Is the study design appropriate and does the work have academic merit?

Yes

Is the work clearly and accurately presented and does it cite the current literature?

Yes

If applicable, is the statistical analysis and its interpretation appropriate?

Yes

Are all the source data underlying the results available to ensure full reproducibility?

Yes

Are the conclusions drawn adequately supported by the results?

Yes

Are sufficient details of methods and analysis provided to allow replication by others?

Yes

Reviewer Expertise:

Rock magnetism, paleomagnetism

I confirm that I have read this submission and believe that I have an appropriate level of expertise to confirm that it is of an acceptable scientific standard.

# Reviewer report: Reviewer response for version 1

**DOI:** 10.21956/openreseurope.21927.r56802

**Published:** 2025-08-28

**Authors:** Tesfaye Kidane

**Affiliations:** 1 Wayne State University, Detroit, Michigan, USA

**Review of the Open Europe paper by SIER et al.** **(Mark J. SIER, Bianca R. Spiering, Mark J. Dekkers, Frits J. Hilgen)**

 The paper entitled “ **Magnetic Mineralogy of the Baringo core (HSPDP-BTB13-1A, Kenya) shows astronomical forcing with implications for retrieving meaningful paleointensity” by Sier et al**, attempts evaluating the potential of the BTB13 sedimentary core from the Baringo Basin, Kenya, to contribute towards the relative paleointensity (RPI) records and improve regional stratigraphic and geochronologic correlation at the scale of East African region.

**General comments**

 The time window (~3.3–2.6Ma), the BTB13-1A sedimentary core covers, overlaps with the ages of various well known hominid-bearing horizons at many localities in the region, including the Baringo in south, and Omo-Turkana, Chew Bahir basins in the North, and potentially further north in Afar depression, hence age constraints on the Baringo core is of great scientific interest.

 Although the original objectives of the authors to contribute to the Relative Paleointensity (RPI) couldn’t be achieved, due to the non-suitability of the studied cores, which are claimed to be dominated by environmental influences, the authors disclosed that the BTB13-1A core preserved a strong astrochronological signal, which can be used as another important correlative tool.

 The astrochronological records of the BTB13 core, which they identified, could potentially be used as a tool for regional correlation and paleoenvironmental reconstruction related to Hominin evolution of the region, including the Omo-Turkana Basin, and further North in the Afar Depression. As a result, the authors have done a good job of identifying and describing the astrochronological signal of the core.  

**Specific comments**

 I would avoid the theoretical aspects of the manuscript in spectral analyses and band filtering and focus on the character of the sedimentary core analyses.

  

1. The introduction needs to be shortened, as it mainly focuses on previous studies on RPI and how that could be used for regional correlations. Because the authors have now tested and known the non-suitability of the core for RPI reconstruction, they rather focus on their main positive contribution, spectral analyses, and astrochronological signal.
2. Could the authors say more about the paradox of having titanomagnetite with single domain (SD) grain sizes as predominant remanence carriers from previous studies (Deino et al., 2006, Deino et al., 2021), and their report of a lack of samples that pass the minimum selection criteria for use of pseudo-Thellier or classic NRM/ARM for RPI analysis.
3. What material characteristics of sediment and sedimentation rate are responsible for elevating NRM/ARM values? Somewhere, it would be helpful to add an explanation of how astrological forcings/fluctuations would result in a material deposition that is characterized by magnetic mineral concentration and grain sizes (rock-magnetic data), with a high NRM/ARM ratio, and low susceptibility.
4. Are there oxygen isotope stratigraphy studies or similar high-quality paleoclimatic and paleoenvironmental studies on the BTB13-1A core to compare with the astrochronological fluctuation reported?  
5. A typo in Page 3, paragraph 2, line 5 (at the end of the line, change “end” to and).
6. When referring to previous magnetostratigraphy work in the region, there are more works to be cited in the neighboring basins to the North – Omo-Turkana basin, such as Kidane et al. 2007, Kidane et al. 2014, and the online stratigraphy chart of Turkana basin ( https://www.turkanastratigraphy.org/strat-chart/main-strat-chart). This is particularly valid if the authors plan to use the tool in other HSPDP sites, which are either structurally linked basins (such as Chew Bahir), or further north of Omo-Turkana.
7. Page 8, last paragraph and last statement going into page 9, 1 ^st^ paragraph, states that NRM/ARM peaks are not an expression of the diatomite formation, but both were an expression of the same driving force. If so, why would one need diatomite in the Northern Awash cores to identify these astrochronological signals?  

The manuscript is well-written, and the authors include some of the leading authorities in paleomagnetism studies focusing on astronomical calibrations. I recommend indexing of the manuscript with minor revisions.

Is the study design appropriate and does the work have academic merit?

Yes

Is the work clearly and accurately presented and does it cite the current literature?

Partly

If applicable, is the statistical analysis and its interpretation appropriate?

Not applicable

Are all the source data underlying the results available to ensure full reproducibility?

Partly

Are the conclusions drawn adequately supported by the results?

Yes

Are sufficient details of methods and analysis provided to allow replication by others?

Partly

Reviewer Expertise:

Structural Geology, tectonics, and paleomagnetism

I confirm that I have read this submission and believe that I have an appropriate level of expertise to confirm that it is of an acceptable scientific standard.

## References

[1] : Precessional forcing of lacustrine sedimentation in the late Cenozoic Chemeron Basin, Central Kenya Rift, and calibration of the Gauss/Matuyama boundary. *Earth and Planetary Science Letters* .2006;247(1-2) : 10.1016/j.epsl.2006.04.009 41-60 10.1016/j.epsl.2006.04.009

[2] : Chronostratigraphy of the Baringo-Tugen Hills-Barsemoi (HSPDP-BTB13-1A) core – 40Ar/39Ar dating, magnetostratigraphy, tephrostratigraphy, sequence stratigraphy and Bayesian age modeling. *Palaeogeography, Palaeoclimatology, Palaeoecology* .2021;570: 10.1016/j.palaeo.2019.109519 10.1016/j.palaeo.2019.109519

[3] : Two normal paleomagnetic polarity intervals in the lower Matuyama Chron recorded in the Shungura Formation (Omo Valley, Southwest Ethiopia). *Earth and Planetary Science Letters* .2007;262(1-2) : 10.1016/j.epsl.2007.07.047 240-256 10.1016/j.epsl.2007.07.047

[4] : Magnetostratigraphy of the Fossil-Rich Shungura Formation, southwest Ethiopia. *Journal of African Earth Sciences* .2014;97: 10.1016/j.jafrearsci.2014.05.005 207-223 10.1016/j.jafrearsci.2014.05.005
